# Health literacy, health perception and related factors among different ethnic groups: a cross-sectional study in southeastern Turkey

**DOI:** 10.1186/s12889-021-11119-7

**Published:** 2021-06-10

**Authors:** Gülhan Yiğitalp, Vasfiye Bayram Değer, Sema Çifçi

**Affiliations:** 1 Department of Nursing, Diyarbakir Atatürk School of Health, Dicle University, Diyarbakır, Turkey; 2 Department of Nursing, Faculty of Health Sciences, Mardin Artuklu University, Mardin, Turkey

**Keywords:** Ethnic group, Health literacy, Health perception, Turkey

## Abstract

**Background:**

Low levels of health literacy are associated with increased hospitalization rates, problems regarding the proper intake of medications, poor general health and increased mortality rates. It is a well-known fact that health literacy differs among ethnic groups and ethnic minorities, in particular, are known to have a low level of health literacy. The present study aimed to reveal the levels of health literacy among different ethnic groups and the affecting factors as well as the relationship between health literacy and health perceptions**.**

**Methods:**

This cross-sectional study was carried out with different ethnic groups (Kurdish, Arab, Turkish and Assyrian origin), between 18 and 65 years old in the province of Mardin in Turkey. The study was conducted with a total of 600 people. The European Health Literacy Scale-Turkish Adaptation (EHLS-TR) and Health Perception Scale (HPS) were used for measurement. Descriptive analysis, Mann Whitney U Test, Kruskal Wallis Test and Spearman correlation were used in the data analysis.

**Results:**

It was found that 80.7% of the participants had relatively low levels of health literacy. The lowest levels of health literacy were among those of Kurdish origin. There were correlations between sufficient levels of health literacy and several factors including being of Assyrian origin, being 50–65 years old, living in a nuclear family, being a secondary school graduate, having a high financial status, being retired, evaluating one’s own health status as good, obtaining health information from healthcare professionals, preferring to visit a state hospital to seek medical assistance first, smoking and drinking alcohol. A positive correlation was also identified between the levels of health literacy and health perception.

**Conclusions:**

It is essential to develop programs to increase health literacy for the public and, in particular, for the ethnic groups that are disadvantaged in many aspects in the context of health literacy.

## Background

The concept of health literacy (HL) is closely linked to conventional literacy and requires knowledge, motivation and competence needed to access, understand, evaluate and use the necessary health information in order for individuals to make health decisions in their daily life, enhance and maintain their quality of life and to prevent disease [1].

Although the levels of HL tend to vary across countries, it is noteworthy that they are generally low. In a study conducted in eight European countries, it was stated that 47.6% of the participants had inadequate and problematic levels of HL. It was reported that the average HL scores differed significantly among the countries and the insufficient HL levels were between 1.8 and 26.9% among these countries. It was also revealed that the Netherlands had the highest HL scores, while Bulgaria had the lowest [2]. Inadequate and problematic HL levels in adults vary between 11.4 and 85.4% in different countries [3–9]. In a national study carried out among adults in Turkey, it was revealed that 64.6% of the participants had inadequate and problematic levels of HL [10]. In another study, it was found that 52.7% of the participants had inadequate and problematic levels of HL [11]. In these studies, different measurement tools related to HL were used. Therefore results may differ.

The fact that HL levels are very low worldwide poses a major health concern. Low levels of HL are associated with increased hospitalization rates, lower use of healthcare, problems regarding the proper intake of medications, difficulty in interpreting labels and health messages, poor general health and increased mortality rates [12]. Additionally, smoking and alcohol abuse [13–15] and low physical activity [16] behaviors which adversely affect health, and high health costs [17], are also associated with low levels of HL.

HL is associated with several demographic factors such as old age, low educational levels, low socioeconomic status and ethnic minorities [10, 11, 16, 18]. Although many researchers have focused on community-based HL, only a few have set out to investigate different ethnic groups. In studies conducted in various countries, it has been reported that HL levels differ among ethnic groups and that ethnic minorities, in particular, have lower HL levels [18–21]. However, the HL levels of ethnic groups in Turkey have not been studied.

Health perception (HP) refers to an individual’s personal beliefs and statements regarding their health status. HP is a subjective term and a strong indicator that reflects the physical, mental and social well-being state of an individual [22]. HP affects the health-seeking behaviors and health responsibility of individuals [23]. HP is also associated with HL, as it is known to increase as HL increases [24–26].

HL has been recognized as a challenging subject in Turkey and is one of the most important issues to be dealt with to any improvement in the health system and to improve quality [27]. Understanding the prevalence of HL is the first step to developing evidence-based interventions to protect and promote public health and healthcare services [28]. Despite the growing interest of researchers in HL in recent years, it is not exactly known whether HL varies according to ethnic differences in Turkey, the explanatory reasons for this and its relationship between HP. Therefore, it is essential to understand the levels of HL among ethnic groups and their relationship with HP in order to address potential health problems and ethnic inequities in health and to implement health promotion strategies.

This study is the first to compare the trilogy of HL, HP and ethnicity. The present study aimed to reveal the relationship between HL and HP as well as evaluate the HL levels and the factors affecting HL in different ethnic groups. In this way, it aimed to fill in the gap in the literature.

## Methods

### Design

This cross-sectional study was conducted in the central district of Artuklu in the province of Mardin, which is located in the Southeastern Anatolia Region of Turkey, between March and June 2019.

### Background information about the study area

Mardin, which has a population of 838,778 people, is a metropolitan city located in Southeastern Turkey. The population of the central district of Artuklu is 178,154 people. Mardin and its surrounding areas host various ethnic and religious communities and elements. Mardin has a much more heterogeneous population in terms of culture and ethnicity compared to any other city in Turkey. Different ethnic groups, consisting mostly of Kurdish, Arab, Turkish and Assyrian people, live together peacefully in Mardin, where the official language is Turkish. The majority of the population in the province of Mardin is of Kurdish origin. The second largest ethnic group is of Arab origin, while the minority group is of Assyrian origin. The ethnic groups of Kurdish, Arab and Turkish origin are Muslims, while the ethnic group of Assyrian origin is Christians [29].

### The study population and sampling

The study population consisted of adult individuals of different ethnic groups aged between 18 and 65 years who live in the central district of Artuklu in Mardin. Snowball and convenience sampling methods were used for the selection of the study sample. In view of the prediction that valid and reliable data could be obtained and as the population of those of Assyrian origin is small and not fully recognized, snowball and convenience sampling method were used to reach this population. After reaching 150 people of Assyrian origin aged between 18 and 65, the same number of people of Turkish, Kurdish and Arab origin living in the same environment were included in the study. By reaching an equal number of people from each ethnic group, the field study was completed with a total of 600 people.

*The inclusion criteria for the study were as follows:* being aged between 18 and 65, being literate in Turkish, volunteering to participate in the study and having communicative skills.

### Data collection tools

Data was collected using a “Personal Information Form”, the European Health Literacy Scale Turkish Adaptation (EHLS-TR) and the Health Perception Scale (HPS).

#### Personal information form

This form was created by the researchers and included questions related to demographic information such as ethnic origin (Kurdish, Arab, Turkish and Assyrian origin), age (18–29, 30–39, 40–49, 50–65), gender (female, male), occupation (housewife, laborer, retired, student, civil servant, tradesman), marital status (married, single), family type (nuclear, extended), educational status (primary school, high school, university or higher), socioeconomic status (fairly high, high, moderate, poor), information regarding any diseases requiring regular medication (yes, no), the source for accessing health information (healthcare professionals, TV/radio, printed press, internet, family members, friends), the type of health facility first applied to in case of a need (family healthcare center, state hospital, private hospital) and the status of smoking and drinking alcohol (yes, no).

#### The European health literacy scale-Turkish adaptation (EHLS-TR)

The scale was developed by the European Health Literacy Research Consortium [30] and the validity and reliability of the Turkish version was performed by Abacıgil et al. [11] It is a self-report scale developed to evaluate the HL of individuals aged 15 years and above. The conceptual framework includes three dimensions related to health (health care, disease prevention and health promotion) and four information processing competencies (accessing, understanding, appraising and applying) related to health-related decision-making and practices. The conceptual framework of the 47-item scale consists of a matrix with 12 sub-dimensions. Each item is evaluated on a 4-point Likert scale, with 1 point = Very hard, 2 points = Hard, 3 points = Easy and 4 points = Very easy. The response ‘I don’t know’ is coded as 5. The total score that can be obtained from the scale is between 47 and 188. For ease of calculation, the total score was standardized to take a value between 0 and 50. The level of health literacy was evaluated in four categories according to the score obtained as (0–25): inadequate health literacy, (> 25–33): problematic health literacy, (> 33–42): sufficient health literacy and (> 42- 50): excellent health literacy. The scale reliability was examined using Cronbach’s alpha coefficient and the construct validity was assessed by principal axis factoring procedure. It was stated that the HLS-TR was a valid and reliable measuring instrument with appropriate psychometric characteristics. A statistically significant and high correlation was found between HLS-TR and sub-dimension scores. The Cronbach’s alpha values of the Turkish version of the scale were found to be 0.95 for total HL, 0.86 for health care, 0.87 for disease prevention and 0.91 for promoting health (11). In the present study, these values were found to be 0.95, 0.81, 0.87 and 0.88, respectively. The EHLS-TR scale was chosen for this study as it is more comprehensive and can be used for international comparisons.

#### Health perception scale (HPS)

This scale was developed by Diamond et al. [31] and its validity and reliability were carried out by Kadıoğlu and Yıldız [32]. There are 15 items and 4 dimensions (control of center, self-awareness, certainty and importance of health) in HPS. It is a five-point Likert type scale. Six items of the scale are positive and 9 items are negative expressions. A minimum of 15 points and a maximum of 75 points can be obtained from the scale. The validity of the scale was evaluated with factor analysis and content-scope validity and reliability were evaluated with item-total score correlation, internal consistency and continuity methods. Kendal W analysis was used for content validity, and Cronbach’s alpha coefficient was used for internal consistency, and Pearson correlation analysis was used for item analysis and test-retest analysis. It was stated that the Turkish version of the scale is a valid and reliable scale. In the study conducted by Kadıoğlu and Yıldız, Cronbach’s alpha values were found to be 0.90 for the control of center dimension, 0.91 for the self-awareness dimension, 0.91 for the certainty dimension and 0.82 for the importance of health dimension. In the present study, these values were found to be 0.80, 0.62, 0.75 and 0.60, respectively. In our study, the Cronbach Alpha value of two dimensions (self-awareness and importance of health) was found to be low. This may be due to our sample being different and our data not being distributed normally. The HLS scale was chosen for this study because it is a scale with proven Turkish validity and reliability in this field.

### Data collection

Data collection was initially started amongst the least known ethnic group, which was those of Assyrian origin. This group was in the minority and their place of residence was not clearly known. Various methods were used to get in touch with these individuals. Governmental institutions, local organizations, congregations, churches and tradesmen were contacted. Thus, Assyrian individuals who met the inclusion criteria were interviewed in their residences and workplaces. Other ethnic groups with the same characteristics living in the same neighborhood were interviewed with the same methods. The participants were informed about the purpose of the study and their consent was obtained. The individuals who accepted agreed to participate in the study and complied with met the inclusion criteria were asked to complete a questionnaire form. Data were collected using the Ppaper and Ppencil interview (PAPI). Those who had difficulty in completing the questionnaire were assisted by the researchers. The data were collected by face-to-face interviews. A total of 64 participants who met the inclusion criteria did not agree to participate in the study and 17 people whose questionnaires forms were not fully completed were excluded from the study. The response average completion time lasted was 20–25 min.

### Data analysis

The study data were analyzed using SPSS 16.0 for Windows (SPSS Inc., Chicago, IL, USA). Descriptive statistics (averages and percentages), normality tests, Mann-Whitney U Test, Kruskal-Wallis Test, multiple regression and Spearman correlation were used in the analysis of the data. Bonferroni-corrected Mann-Whitney U test was used as a post-hoc procedure to determine the source of the difference as a result of the Kruskal-Wallis Test. The Cronbach’s alpha coefficient was used in the analysis of the internal consistency of the scales. All the findings were tested at a significance level of 0.05.

### Ethical approval

This study was prepared in accordance with the guidelines of the Helsinki Declaration. The study was approved by Non-Interventional Ethics Committee (27.11.2018, 2018 / 1–1) of Mardin Artuklu University. Necessary legal permissions were obtained from the relevant institution. İnformed consent was obtained from all participants who volunteered to participate in the study.

## Results

It was determined that among the participants, 36.8% of them included in the study were aged between 18 and 29 years. 58.8% of the participants were male and 63.2% of them were married. 54.3% of the participants smoked, while 27.7% of them drank alcohol.

In the study, there was a statistically significant difference between the ethnic groups in terms of age, marital status, family type, educational status, economic status and occupation (*p* < 0.05) (Table 1).

**Table 1:** The relationship between ethnic origin and demographic variables

Characteristics	Turkish origin n (%)	Kurdish origin n (%)	Assyrian origin n (%)	Arab origin n (%)	Total n (%)	χ^2^ p
Age						
18–29	72 (32.6)	41 (18.6)	20 (9.0)	88 (39.8)	221 (100.0)	102.43
30–39	44 (20.6)	72 (33.6)	58 (27.1)	40 (18.7)	214 (100.0)	0.000
40–49	28 (18.7)	27 (23.7)	44 (38.6)	15 (13.2)	114 (100.0)	
50–65	6 (11.8)	10 (19.6)	28 (54.9)	7 (13.7)	51 (100.0)	
Gender						
Female	57 (23.1)	58 (23.5)	68 (7.5)	64 (25.9)	247 (100.0)	2.22
Male	93 (26.3)	92 (26.1)	82 (23.2)	86 (24.4)	353 (100.0)	.527
Marital status						
Married	84 (22.2)	99 (26.1)	112 (29.6)	84 (22.2)	379 (100.0)	15.66
Single	66 (29.9)	51 (23.1)	38 (17.2)	66 (29.9)	221 (100.0)	0.001
Family type						
Nuclear	48 (23.5)	37 (18.1)	94 (46.1)	25 (12.3)	204 (100.0)	81.105
Extended	102 (25.8)	113 (28.5)	56 (14.1)	125 (31.6)	396 (100.0)	0.000
Educational status						
Primary school	31 (13.5)	52 (22.7)	99 (43.2)	47 (20.5)	229 (100.0)	
High School	80 (32.0)	62 (24.8)	35 (14.0)	73 (29.2)	250 (100.0)	73.90
University or higher	39 (32.2)	36 (29.8)	16 (13.2)	30 (24.8)	121 (100.0)	0.000
Economic status						
Fairly high	22 (47.8)	21 (45.7)	3 (6.5)	0 (0.0)	46 (100.0)	82.062
High	84 (23.7)	75 (21.1)	119 (33.5)	77 (21.7)	355 (100.0)	0.000
Moderate	44 (23.3)	50 (26.5)	23 (12.2)	72 (38.1)	189 (100.0)	
Poor	0 (0.0)	44 (0.0)	5 (50.0)	1 (10.0)	10 (100.0)	
Occupation						
Housewife	20 (16.4)	25 (20.5)	48 (39.3)	29 (23.8)	122 (100.0)	
Laborer	26 (22.4)	35 (30.2)	12 (10.3)	43 (37.1)	116 (100.0)	
Retired	3 (20.0)	3 (20.0)	4 (26.7)	5 (33.3)	15 (100.0)	139.714
Student	37 (45.1)	7 (8.5)	4 (4.9)	34 (41.5)	82 (100.0)	0.000
Civil servant	33 (35.1)	39 (41.5)	8 (8.5)	14 (14.9)	94 (100.0)	
Tradesman	31 (18.1)	41 (24.1)	74 (43.3)	25 (14.6)	171 (100.0)	

A statistically significant difference was found between ethnic origin and HL levels (*p* = 0.000). In the categorical evaluation (inadequate, problematic, sufficient, excellent health literacy), 80.7% of all of the participants included in the study had limited (inadequate and problematic) HL levels. While most of the participants of Kurdish origin (85.3%) had inadequate HL levels, those of Assyrian origin had the most sufficient (46.7%) levels (Table 2).

**Table 2:** HL levels of the ethnic groups

Ethnic origin	Inadequate n (%)	Problematic n (%)	Sufficient n (%)	Excellent n (%)	Total n (%)	χ2 p
Turkish origin	54 (36.0)	92 (61.3)	4(2.7)	0 (0.0)	150 (100.0)	
Kurdish origin	128 (85.3)	21 (14.0)	1 (0.7)	0 (0.0)	150 (100.0)	324.09
Assyrian origin	14 (9.3)	63 (42.0)	70 (46.7)	3 (2.0)	150 (100.0)	0.000
Arab origin	20 (13.4)	92 (61.3)	38 (25.3)	0 (0.0)	150 (100.0)	
Total HL	216 (36.0)	268 (44.7)	113 (18,8)	3 (0.5)	600 (100.0)	

There was a statistically significant relationship between ethnic origin, age, family type, socioeconomic status, the source for accessing health information, smoking and drinking alcohol and HP levels (*p* < 0.05). The levels of HP of those of Assyrian origin, those aged between 50 and 65, those living in a nuclear family, those with high socioeconomic status, those who received health information mostly from healthcare professionals, those who consumed alcohol and non-smokers were higher than the other groups. There was a significant relationship between all the variables and HL levels except for the following variables: a disease requiring regular use of medication, gender and marital status. The data revealed that the participants with high HL were likely to be of Assyrian origin, be between the ages of 50–65, be living in a nuclear family, be secondary school graduates, have a high socioeconomic status, be retired, evaluate their health status as good, obtain health information from healthcare professionals, apply to the state hospitals in emergencies, smoke and drink alcohol (*p* < 0.05), (Table 3).

**Table 3:** Comparison of HP and HL scale scores in terms of sociodemographic characteristics

Ethnic origin	HP scale scores					HL scale scores			
	N	X̄±SS	χ2/z	p	Diff.***	X̄±SS	χ2/z	p	Diff.***
^a^Turkish origin	150	41.78 ± 2.16			a-b, a-c,	26.24 ± 2.49			a-b, a-c,
^b^Kurdish origin	150	41.00 ± 2.20	117.013*	0.000	a-d, b-c,	23.04 ± 2.17	315.934*	0.000	a-d, b-c,
^c^Assyrian origin	150	45.28 ± 4.74			b-d, c-d	32.36 ± 5.49			b-d, c-d
^d^Arab origin	150	42.00 ± 3.49				30.25 ± 4.08			
Age									
^a^18-29	221	42.50 ± 3.38				28.23 ± 4.97			
^b^30-39	214	42.45 ± 3.64	8.564*	0.036	a-d, b-c,	27.09 ± 5.12	14.249*	0.003	a-b, b-c,
^c^40-49	114	43.50 ± 4.31			b-d,	28.26 ± 5.06			b-d
^d^50 -65	51	43.58 ± 3.24				29.89 ± 6.40			
Gender									
Female	247	42.73 ± 3.77	-.357**	0.72		28.22 ± 5.26	-.974**	0.328	
Male	353	42.79 ± 3.61				27.80 ± 5.19			
Marital status									
Married	379	42.95 ± 3.62	-1.753**	0.080		28.05 ± 5.38	-.221**	0.825	
Single	221	42.44 ± 3.76				27.83 ± 4.95			
Family type									
Nuclear	204	43.49 ± 4.38	-3.446**	0.001		29.57 ± 5.97	-4.669**	0.000	
Extended	396	42.39 ± 3.20				27.15 ± 4.59			
Educational status									
^a^Primary school	77	43.31 ± 4.47	3.569*	0.312		28.74 ± 5.22	9.411*	0.024	
^b^Secondary school	152	43.25 ± 4.13				28.90 ± 5.73			
^c^High school	250	42.31 ± 2.81				27.38 ± 4.68			a-c, b-c
^d^University or higher	121	42.76 ± 4.01				27.53 ± 5.45			
Economic status									
^a^Fairly high	46	41.69 ± 3.16				24.70 ± 2.61			
^b^High	355	43.09 ± 3.53	14.893*	0.002	a-b, b-c	28.77 ± 5.32	31.144*	0.000	a-b, a-c,
^c^Moderate	189	42.41 ± 4.01				27.23 ± 4.92			b-c
^d^Poor	10	42.70 ± 3.30				28.69 ± 8.51			
Occupation									
^a^ Housewife	122	42.98 ± 4.01				28.89 ± 5.39			
^b^Laborer	116	42.54 ± 3.38				28.06 ± 5.05			
^c^Retired	15	42.60 ± 2.41	8.436*	0.134		29.74 ± 6.15	28.350*	0.000	a-e, b-e,
^d^Student	82	42.06 ± 3.49				28.29 ± 4.54			c-e, d-e,
^e^ Civil servant	94	42.22 ± 2.77				25.55 ± 3.98			f-e
^f^Tradesman	171	43.42 ± 4.14				28.28 ± 5.68			
Evaluation of health status									
^a^Great	28	42.14 ± 6.93				26.10 ± 5.72			
^b^Good	448	42.88 ± 3.49	7.136*	0.068		28.42 ± 4.95	27.473*	0.000	a-b, b-c
^c^Moderate	114	42.57 ± 3.30				26.73 ± 5.50			
^d^Poor	10	41.50 ± 3.02				27.40 ± 8.94			
Any disease requiring regular medication									
Yes	90	42.16 ± 3.18	-1.552**	0.121		27.51 ± 5.55	-1.48**	0.251	
No	510	42.87 ± 3.75				28.05 ± 5.16			
The source from which health information is obtained									
^a^Healthcare professionals	248	43.35 ± 4.24	21.126*			29.73 ± 4.46	99.12*		
^b^TV/Radio	136	42.52 ± 3.13			a-d, b-d,	26.67 ± 5.70			a-d, b-d,
^c^Printed press	43	41.90 ± 2.57		0.001	c-d, e-d,	25.78 ± 5.09		0.000	c-d, e-d,
^d^Internet	97	43.32 ± 3.39			f-d	28.58 ± 5.23			f-d
^e^Family members	46	41.30 ± 2.51				25.06 ± 4.11			
^f^Friends	32	41.18 ± 3.49				25.02 ± 4.80			
The type of health facility first applied to in case of a need									
^a^Family healthcare center	142	42.20 ± 3.29				25.32 ± 3.78			
^b^State hospital	406	42.77 ± 3.43	5.764*	0.056		28.80 ± 5.32	49.073*	0.000	a-b, a-c
^c^Private hospital	52	44.30 ± 5.65				28.75 ± 5.49			
Smoking status									
^a^Still smoking	326	43.09 ± 3.51				28.94 ± 5.43			
^b^Given up	158	41.67 ± 2.53	20.456*	0.000	a-b, b-c	26.57 ± 4.09	24.188*	0.000	a-b, a-c
^c^Never smoked	116	43.35 ± 4.96				27.12 ± 5.46			
Drinking alcohol									
Yes	166	44.56 ± 4.67	-7.275**	0.000		31.11 ± 5.82	-8.339**	0.000	
No	434	42.08 ± 2.95				26.77 ± 4.43			

* Kruskal Wallis Test

**Mann-Whitney U Test

*** Bonferroni-corrected Mann-Whitney U test was used as post-hoc procedure to determine the source of the difference as a result of the Kruskal-Wallis Test

^a-f^ Post-hoc analysis

A statistically significant correlation was determined between the HL and HP total scores of the participants. There was a moderately positive (*r* = 0.454 *p* = 0.000) correlation between the HL and HP scores (Table 4).

**Table 4:** The correlations between the mean scores of the HL and the mean scores of the HP

Scales^a^	HL scale	HP scale
HL scale	1	*r* = 0.454^b^
		*p* = 0.000
HP scale	*r* = 0.454^b^ *p* = 0.000	1

^a^Spearman correlation test was used

^b^ The correlations are significant at 0.01 level

## Discussion

The results showed that those of Assyrian origin had the highest average HL score, while those of Kurdish origin had the lowest score. When the proportion of people with limited (inadequate and problematic) HL among the ethnic groups are considered (ranged from 51.3 to 99.3%), it is obvious that the levels of HL pose an important public health problem for the region. Although these ethnic groups live in the same cultural environment, they have different characteristics in terms of religion, beliefs and various cultural aspects. As such, their access, understanding and use of health information may differ and this study has revealed ethnic group with Kurdish origin has the highest proportion of people with inadequate HL.

To the researchers’ knowledge, the present study is the first to provide a comparison of HL levels among individuals of different ethnic backgrounds living in Turkey. Therefore, the results of the present study regarding HL levels in different ethnic groups could not be compared with the findings of existing national studies. Compared to the other community-based studies conducted in Turkey [10, 11, 33, 34], it is noteworthy that the inadequate HL levels in the present study were significantly higher. Considering the connection between HL and fundamental literacy, this could be explained by the fact that the literacy levels are lower in southeastern Turkey compared to other regions [35]. The findings of the studies conducted among ethnic groups in many countries have revealed different conclusions regarding the levels of HL. For example, in a study conducted with women of different ethnicities in Taiwan, one third of the women had an inadequate HL level and the HL levels of Taiwanese women were found to be higher than those of ethnic minorities [19]. In a study conducted with young adults in Amsterdam, the HL of 17.0% of the participants was found to be low and young Dutch adults were determined to have scored higher in terms of HL compared to other ethnic groups [20]. In a study conducted among refugees in Sweden, it was shown that three-fifths of the participants had inadequate and problematic HL [36]. In a study conducted in European countries, it was observed that approximately half of the individuals had inadequate and problematic HL [2]. Results in this study were also compared with studies using different HLS. This can make a difference in comparing results. The ethnic groups included in the present study differ from those in other countries as they have been living in this country together for a long time. Although those of Assyrian origin, especially those living in Mardin, are numerically less than other ethnic groups and differ from the other ethnic groups in terms of religion, beliefs and various cultural aspects, they have many common characteristics (eating and drinking, clothing, social life, etc.) with other cultures. Therefore, the HL levels of ethnic groups in Mardin may be affected differently.

The results of this study showed that the levels of HL were associated with several variables such as age, family type, educational status, economic status, occupation, and evaluation of one’s health status. The data revealed that the participants with high HL were likely to be of Assyrian origin, be between the ages of 50–65, be living in a nuclear family, be secondary school graduates, have a high socioeconomic status, be retired, evaluate their health status as good, obtain health information from healthcare professionals, apply to the state hospitals in emergencies, smoke and drink alcohol. The results of many studies conducted among adult individuals in different countries have shown that those more likely to have low HL levels are older adults, unemployed people, individuals with low educational and low-income levels [37–39]. Furthermore, it has been revealed that those who evaluate their own health as bad also have a low level of HL [5, 36, 40]. These findings, indicating that some social groups have low levels of HL, seem to show that there are various social factors involved in HL. The findings in the literature are generally similar to those of the present study. However, contrary to the findings in the literature, the HL scores of the elderly (aged between 50 and 65 years) were higher in the present study. A similar result was also obtained in a study conducted in Japan [6]. Furthermore, another result that was different from those in the literature was that those with a low education level had higher HL. In this study, the fact that individuals of Assyrian origin had the highest HL scores and tended to be older might have contributed to the high HL scores of the elderly individuals. Individuals of Assyrian origin might have their specific ways to achieve and use information on health more, which might also contribute to higher HL scores.

It should be noted that the HL scores among smokers and drinkers of alcohol were surprisingly higher than non-smokers and non-drinkers. Contrary to this finding, several studies in the literature have indicated a negative relationship between HL and smoking [13, 14, 41]. Garcia-Codina et al. [40] reported that those who drank less alcohol had sufficient HL levels compared to heavy drinkers and non-drinkers. Furthermore, Amoah et al. [15] found that poor functional HL was associated with alcohol abuse. There are limited studies revealing the relationship between HL and smoking and alcohol abuse. In order to verify this relationship and explain this unexpected result of the present study, it is significantly important to conduct more extensive research that also reveals to explore the causal relationships between smoking and drinking alcohol abuse and HL.

It is important for all individuals to possess the skills that enable them to access and select reliable sources of information. The efforts to seek information with these skills are central to the processes by which HL affects health outcomes. In a study conducted in the USA, it was stated that the participants mostly considered health professionals as a reliable source of health information [42]. In other studies, it has also been shown that health professionals are the most preferred source of information [43–45]. Health professionals are always among the top three sources sought for health information and the first reliable source of health information in Turkey [46]. These findings are consistent with the results of the present study.

Another important result of this study was that as HL levels increased, the HP levels of individuals increased. HP is associated with individuals’ health protection and development behaviors, self-care management, and levels of HL in general [47]. Although the studies investigating the relationship between HL and HP are limited, some studies have reported results supporting the positive relationship between HL and HP [24, 26, 47–50]. In this context, it can be suggested that individuals with sufficient levels of HL are more likely to seek health information, protect and promote their health, and look for solutions in cases of illness.

This study is the first step to conducting future studies in Turkey measuring the levels of HL among ethnic groups with larger sample size. In the light of these data, programs to increase HL should be developed in Mardin, especially for disadvantaged groups. In order to increase the HL level of the public to the desired level, it is important to include HL lessons in the curriculums at schools. Also, in-service trainings must be organized for healthcare employees to develop programs to increase the HL levels of the society. These interventions will likely help improve the health literacy of individuals. However, more quantitative and qualitative research is required to investigate HL levels and the factors affecting the levels of the ethnic groups living in Mardin and to identify strategies to tackle the challenges.

### Research limitations

The limitations of this study are as follows: 1) As the study was carried out in only a certain geographical region, the results cannot be generalized to the whole of the Turkish population, 2) As the improbable sampling method was used, there are certain limitations in generalizing the findings in the study group, 3) The answers given to the questions may include recall bias as the study was conducted as a cross-sectional design. 4) The total scores of the scales were evaluated while the sub-scales were not compared, which can lead to poorer predictions of HL and HP levels. 5) Only people who understood Turkish were included in the study and disadvantaged groups who did not speak Turkish were not included. Future studies may consider exploring the HL of these ethnic groups. However, the fact that there are no studies in the literature evaluating ethnic groups in terms of HL and HP is the strength of the present study.

## Conclusion

The findings showed that inadequate levels of HL were dramatically high among the ethnic groups in general and that those of Kurdish origin were the most disadvantaged group. It was considered striking that older people, those with low educational levels and those who smoke and drank alcohol had higher HL scores. In addition, a moderately positive relationship was determined between HL and HP. This study provided insights into the HL status of ethnic groups in Turkey. Further studies are required to identify strategies to help improve the HL of these ethnic groups.

## Data Availability

The datasets used and/or analyzed during the current study are available from the corresponding author on reasonable request.

## Abbreviations

EHLS-TR: The European Health Literacy Scale-Turkish Adaptation
HL: Health Literacy
HP: Health perception
HPS: Health Perception Scale
SPSS: Statistical Package for Social Sciences
USA: United States of America
